# Prophylactic and Therapeutic Anti-Hyperglycemic Effects of Heat-Killed *Mycobacterium aurum* in STZ-Induced Diabetic Mice

**DOI:** 10.3390/nu18111652

**Published:** 2026-05-22

**Authors:** Ali Ali, Hanin-Khaula Hakam, Alaa Eter, Samer Bazzi, Amani Chahine, Charles Akle, Georges M. Bahr, Karim S. Echtay

**Affiliations:** 1 Department of Biomedical Sciences, Faculty of Medicine and Medical Sciences, University of Balamand, 33 Amioun, Al-Koura, Tripoli P.O. Box 100, Lebanon; ali.m.ali@std.balamand.edu.lb (A.A.); alaa.eter@std.balamand.edu.lb (A.E.); amani.chahine@fty.balamand.edu.lb (A.C.); 2 Immune Boost Clinic Limited, Shenstone Strathclyde, Bridgetown BB11078, St Michael, Barbados; cakle@me.com

**Keywords:** postbiotic, functional food, *Mycobacterium aurum*, metabolic disease, diabetes hyperglycemia, uncoupling proteins, glucose transporters, antioxidant defense

## Abstract

**Background/Objectives**: Exploiting the metabolic properties of postbiotics is a novel strategy for managing metabolic disorders, including diabetes. Inactivated microorganisms, a major class of postbiotics, improve glycemic control in preclinical and clinical studies. Here, we examined whether heat-killed (HK) *Mycobacterium aurum* (*M. aurum*) exerts prophylactic or therapeutic anti-hyperglycemic effects in diabetic mice. **Methods**: Diabetes was induced in male BALB/c mice by streptozotocin (STZ; 150 mg/kg) injection. HK *M. aurum* (1 mg) was given orally (three prophylactic doses before STZ) or intradermally (six weekly therapeutic doses after STZ). We assessed glycemic parameters, serum *C*-peptide/insulin (ELISA), and tissue protein expression (Western blot). **Results**: Neither route altered body weight or glucose homeostasis in non-diabetic mice. In STZ-diabetic mice, oral prophylactic treatment significantly attenuated hyperglycemia (39–60% reduction weeks 5–8 post-STZ) and showed a trend toward improved serum *C*-peptide, but did not affect dysregulated expression of skeletal muscle (SM), hepatic, pancreatic and renal proteins involved in glucose transport (GLUT2, GLUT4, and SGLT2), glycolysis (α-LDH), mitochondrial uncoupling (UCP2 and UCP3), and antioxidant defense (CAT). Therapeutic intradermal administration significantly decreased blood glucose (~30% at week 5, ~40% at week 6) and modestly enhanced insulin secretion. Hepatic UCP2 and α-LDH and SM UCP3 protein levels were normalized toward non-diabetic levels, whereas hepatic GLUT2 and SM GLUT4 remained largely unchanged. These correlative findings suggest effects independent of insulin-dependent glucose transport, but do not demonstrate direct functional improvement in mitochondrial or redox status. **Conclusions**: HK *M. aurum* exerts partial anti-hyperglycemic effects in STZ-induced diabetic mice, but the associated protein changes require functional validation before its role as a postbiotic in β-cell dysfunction can be established.

## 1. Introduction

Diabetes mellitus (DM) is a chronic, complex metabolic disorder characterized by persistent hyperglycemia resulting from defects in insulin secretion, insulin action, or both. DM is primarily classified into Type 1 diabetes (T1D), an autoimmune disease characterized by the destruction of insulin-producing pancreatic beta cells, and Type 2 diabetes (T2D), which arises mainly from insulin resistance and relative insulin deficiency [1]. The global burden of diabetes is increasing at an alarming rate, posing a major public health challenge worldwide [2].

Current therapeutic strategies for managing diabetes include lifestyle interventions, oral and injectable pharmacological agents, and insulin therapy. Although such interventions can help manage hyperglycemia, they are often associated with limitations such as adverse effects, high cost, limited accessibility, and an inability to halt disease progression [3,4]. These challenges highlight the urgent need for innovative, but safe, therapeutic and preventive approaches that can modulate the underlying pathophysiology of diabetes more effectively. In recent years, increasing attention has been directed towards the potential usage of postbiotics in the management of metabolic diseases such as diabetes [5,6]. Inanimate microorganisms are a type of postbiotic prepared using methods such as heat, pressure, or irradiation, thus influencing their functional properties [7]. Murine studies have shown that inactivated multi-species probiotics, *Bifidobacterium longum*, *Bifidobacterium animalis*, and *Akkermansia muciniphila* (*A. muciniphila*), reduce fasting blood glucose, improve glucose tolerance, protect pancreatic cells, and enhance insulin sensitivity, often via gut microbiota modulation and reduced inflammation [8,9,10]. Additionally, two human trials confirmed that inanimate *Lacticaseibacillus casei* attenuates postprandial glucose responses, while pasteurized *A. muciniphila* lowers plasma insulin and improves insulin sensitivity in overweight/insulin-resistant individuals, with no adverse effects [11,12]. In this context, heat-killed (HK) mycobacteria may serve as a whole-cell postbiotic with potential anti-diabetic properties. Previous work from our laboratory has demonstrated that multiple prophylactic intradermal (ID) injections of HK *Mycobacterium aurum* (*M. aurum*) significantly reduced hyperglycemia in streptozotocin (STZ)-induced diabetic mice. The same treatment also improved glucose utilization, mitochondrial function, and oxidative stress in the liver and skeletal muscle (SM) tissues [13]. *M. aurum*, also known as *Mycolicibacterium aurum* Aogashima, is a non-pathogenic environmental mycobacterium that is currently available as a commercially available, HK whole-cell preparation formulated in a capsule form and is marketed as a dietary supplement for the management of chronic inflammation, anxiety, and stress [14]. In terms of safety, oral administration of multiple doses of HK *M. aurum* was reported to be well tolerated in rats, with no adverse effects on weight or toxicity, and lacks genomic features associated with pathogenicity, toxigenicity, or transferable antibiotic resistance [15]. More recently, it was demonstrated that intragastric administration of HK *M. aurum* stabilizes the intestinal microbiome and protects against stress-induced exacerbation of dextran sulfate sodium colitis in mice [16].

We examined whether HK *M. aurum* could prevent or treat diabetes in BALB/c mice rendered diabetic with a single high dose of STZ. The high-dose STZ model induces severe insulin deficiency via β-cell destruction and is useful for studying interventions in an insulin-poor state, though it does not fully replicate the autoimmune etiology of human T1D [17,18]. Findings from this model should not be directly extrapolated to T2D or insulin-resistant states without further validation. To test its protective effects, *M. aurum* was given orally prior to diabetes induction, allowing evaluation of route independence and practical translational use. For therapeutic assessment, *M. aurum* was administered intradermally immediately post-STZ injection so as to determine its capacity to reverse metabolic dysfunction.

In both mouse experimental setups, we monitored body weight, blood and urine glucose levels, serum insulin and *C*-peptide levels, and protein expression levels of glucose transporters and oxidative stress-related enzymes, mainly in the liver and SM tissues that are particularly vulnerable to hyperglycemia-driven mitochondrial dysfunction [19,20,21]. We undertook this two-arm experimental approach to explore how HK *M. aurum* might influence immune and metabolic pathways that are relevant to diabetes prevention and care. The knowledge gained from this work could support the development of a safe, practical, and effective postbiotic that can be incorporated into functional foods and work alongside current diabetes management strategies.

## 2. Materials and Methods

### 2.1. Animals

A total of 127 adult male BALB/c mice (aged between 4 and 8 weeks old; weighing 19–30 g) were provided by the University of Balamand animal care facility. Mice were maintained under controlled room conditions (22 ± 2 °C; 12 h light/dark cycle) and fed regular chow ad libitum. Each mouse group was acclimated for 1 week prior to the start of the experiments. All mouse-related procedures were approved by the University of Balamand Institutional Animal Care and Use Committee (protocol#: IACUC02/2025; 5 March 2025).

### 2.2. Preparation of HK M. aurum and STZ

A sterile whole-cell suspension of HK *M. aurum* DSM 33539 (stock concentration: 200 mg/mL in distilled water) was manufactured by autoclaving at 121 °C for 15 min (kindly supplied by Immune Boost Clinic Limited, Bridgetown, Barbados). A subdilution of HK *M. aurum* [1 mg in 100 µL of borate-buffered saline (BBS)] was freshly prepared prior to each use in animal experiments. According to an earlier study [13], treatment of mice with 3 doses of HK *M. aurum* (1 mg/100 µL of BBS) was safe and demonstrated an anti-diabetic potential for the mycobacterial preparation. Consequently, this dosage was used in the current study. Streptozotocin (STZ; Sigma-Aldrich, St. Louis, MO, USA) solution was freshly prepared in ice-cold citrate buffer (CB; pH 4.5) and injected intraperitoneally into mice (at a dosage of 150 mg/kg body weight) within 30 min of solution preparation.

### 2.3. Experimental Design for Prophylactic Study

To evaluate the prophylactic anti-diabetic potential of orally administered HK *M. aurum*, mice were randomly divided into four experimental groups that were age-matched (Supplementary Figure S1). The control group (BBS + CB) received 3 oral doses of BBS (100 µL) at weeks −6, −4, and −2 pre-CB injection, followed by intraperitoneal (IP) injection of CB (100 µL) at week 0. The second group (Ma + CB) received 3 oral doses of HK *M. aurum* (1 mg/100 µL BBS) at weeks −6, −4, and −2 pre-CB injection, followed by IP injection of CB (100 µL) at week 0. The third group (BBS + STZ) received 3 oral doses of BBS (100 µL) at weeks −6, −4, and −2 pre-STZ injection, followed by IP injection of STZ (150 mg/kg body weight in 100 µL CB) at week 0. The fourth group (Ma + STZ) received 3 oral doses of HK *M. aurum* (1 mg/100 µL BBS) at weeks −6, −4, and −2 pre-STZ injection, followed by IP injection of STZ (150 mg/kg body weight in 100 µL CB) at week 0.

### 2.4. Experimental Design for Therapeutic Study

To investigate the potential anti-diabetic therapeutic effects of ID administration (at the base of the tail) of HK *M. aurum*, mice were randomly allocated into four groups, which were age-matched (Supplementary Figure S2). The control group (CB + BBS) received a single IP injection of CB (100 µL) at week 0, followed by 6 ID injections of BBS (100 µL) starting on day 1 post-CB injection and over a period of 6 weeks. The second group (CB + Ma) received a single IP injection of CB (100 µL) at week 0, followed by 6 ID injections of HK *M. aurum* (1 mg/100 µL BBS) starting on day 1 post-CB injection and over a period of 6 weeks. The third group (STZ + BBS) received a single IP injection of STZ (150 mg/kg, 100 µL) at week 0, followed by 6 ID injections of BBS (100 µL) starting on day 1 post-STZ injection and over a period of 6 weeks. The fourth group (STZ + Ma) received a single IP injection of STZ (100 µL) at week 0, followed by 6 ID injections of HK *M. aurum* (1 mg/100 µL BBS) starting on day 1 post-STZ injection and over a period of 6 weeks.

### 2.5. Measurement of Body Weight, Blood and Urine Glucose Levels

Body weight and fasting blood and urine glucose levels were recorded on a weekly basis throughout the duration of the animal studies. Mice were fasted for 4 h prior to each blood collection. Body weight was recorded using an electronic balance. Blood samples were collected from mice that were fasted for 4 h through pricking their tail vein using a 21 G needle. Fasting blood glucose levels of mice were measured using a glucometer and strips (Rightest GM260; Bionime, Taichung, Taiwan). Urine glucose levels of mice were assessed by urine analysis reagent strips (INsight Expert, Acon Laboratories, San Diego, CA, USA).

### 2.6. Quantification of Serum C-Peptide and Insulin Levels

At week 6 or 8 post-CB/-STZ injection, mice were euthanized by cervical dislocation performed by trained personnel, and blood was collected via cardiac puncture into serum separator tubes. After blood clotting for 30 min at room temperature (RT), samples were centrifuged at 1500× *g* for 10 min at 4 °C, and serum was stored at −80 °C for later analyte analysis. Serum *C*-peptide and insulin levels were quantified using commercial linked immunosorbent assay (ELISA) kits according to the manufacturer’s instructions (Invitrogen, Thermo Fisher Scientific, Waltham, MA, USA). All samples and standards were analyzed in duplicate. *C*-peptide kit sensitivity was 9.38 pg/mL, while its standard range was 15.63–1000 pg/mL; the insulin kit sensitivity was 5 μIU/mL, and its standard range was 6.25–400 μIU/mL.

### 2.7. Tissue Collection, Protein Lysate Preparation and Quantification

At week 6 or 8 post-CB/-STZ injection, mice were euthanized by cervical dislocation and tissues (SM, liver, pancreas, and kidney) were excised, rinsed in cold phosphate-buffered saline (PBS), snap-frozen in liquid nitrogen, and stored at −80 °C. Frozen tissues were homogenized in lysis buffer, incubated on ice for 45 min with intermittent mixing, and centrifuged at 14,000 rpm for 10 min at 4 °C. Supernatants were collected and stored at −80 °C for later protein quantification and Western blotting. Protein concentrations in each tissue sample were determined using the Biuret method. Absorbance was measured at 540 nm, and concentrations were calculated from a standard curve generated with bovine serum albumin (BSA).

### 2.8. Western Blot

Total protein lysates from SM, liver, pancreas, or kidney harvested from different groups of mice were separated on 12% SDS-PAGE gel and transferred onto polyvinylidene difluoride (PVDF) membrane. The membrane was blocked for 1 h at RT in 5% BSA in tris-buffered saline (TBS) containing 1% Tween 20 (TBS-T) and incubated overnight at 4 °C with the appropriate primary antibody [anti-uncoupling protein 2 (UCP2), anti-uncoupling protein 3 (UCP3), anti-glucose transporter 2 (GLUT2), anti-GLUT4, anti-alpha lactate dehydrogenase (α-LDH), anti-catalase (CAT), or anti-sodium-glucose cotransporter 2 (SGLT2)] (Invitrogen, Thermo Fisher Scientific). Following incubation with the primary antibody, the membrane was washed 5× (10 min each) with TBS-T and then incubated with the corresponding horseradish peroxidase (HRP)-conjugated secondary anti-mouse IgG (Invitrogen, Thermo Fisher Scientific), anti-goat IgG (Abcam, Cambridge, UK), or anti-rabbit IgG (Novus Biologicals, Littleton, CO, USA) antibody for 90 min at RT under gentle agitation. Afterward, the membrane was washed again 5× (5 min each) with TBS-T at RT and then incubated with Clarity Western ECL substrate (Bio-Rad Laboratories, Hercules, CA, USA)) for 5 min at RT. Protein bands were finally visualized using the Bio-Rad ChemiDoc imaging system, and densitometric analysis was performed with Image Lab software (version 5.2.1; Bio-Rad). All membranes were at least stripped once and re-probed again with anti-glyceraldehyde-3-phosphate dehydrogenase (GAPDH) or anti-β-actin antibody for loading control. Exceptionally, two membranes were stripped twice using stripping buffer; one membrane was used sequentially to assess SM GLUT4, UCP3, and β-actin expression (prophylactic arm), while the other was used for SM GLUT4, CAT, and GAPDH expression (therapeutic arm). Briefly, membranes were stripped using a mild stripping buffer (1.5% glycine, 0.1% SDS, 1% Tween 20; pH 2.2) for 15 min at RT with gentle agitation. Membranes were then re-blocked and incubated with the next primary antibody as described above.

### 2.9. Statistical Analysis

Data were analyzed using GraphPad Prism v9.1 (GraphPad Software, San Diego, CA, USA). Results are expressed as mean ± standard error of the mean (SEM). Statistical differences in body weight, blood and urine glucose data of different mouse groups were analyzed via mixed effect model with repeated measurements, followed by Tukey’s post hoc multiple comparison test. One-way ANOVA followed by Fisher’s LSD post hoc test was applied to evaluate differences in tissue protein expression, serum *C*-peptide and insulin levels of different mouse groups. An independent unpaired *t*-test was performed to compare difference in body weights and blood glucose levels between untreated and *M. aurum*-treated non-diabetic mouse groups. A *p*-value of <0.05 was considered statistically significant. Schematic figures were created with Biorender.com.

## 3. Results

### 3.1. Prophylactic Oral Administration of HK M. aurum Alleviates Hyperglycemia in STZ-Induced Diabetic Mice

Before conducting an evaluation of the prophylactic anti-hyperglycemic efficacy of HK *M. aurum* in STZ-induced diabetic mice, we determined whether oral administration of the HK mycobacterial preparation to non-diabetic mice would affect their body weight, blood and urine glucose levels. Over a period of 15 weeks, there were no significant differences in the body weights and blood glucose levels between the non-diabetic mice pretreated with 3 doses of 1 mg of HK *M. aurum* and the non-diabetic mice pretreated with 3 doses of BBS vehicle, as both agents were administered orally 2 weeks apart (at weeks −6, −4, and −2 pre-CB injection) (Supplementary Figure S3).

**Figure 1:**
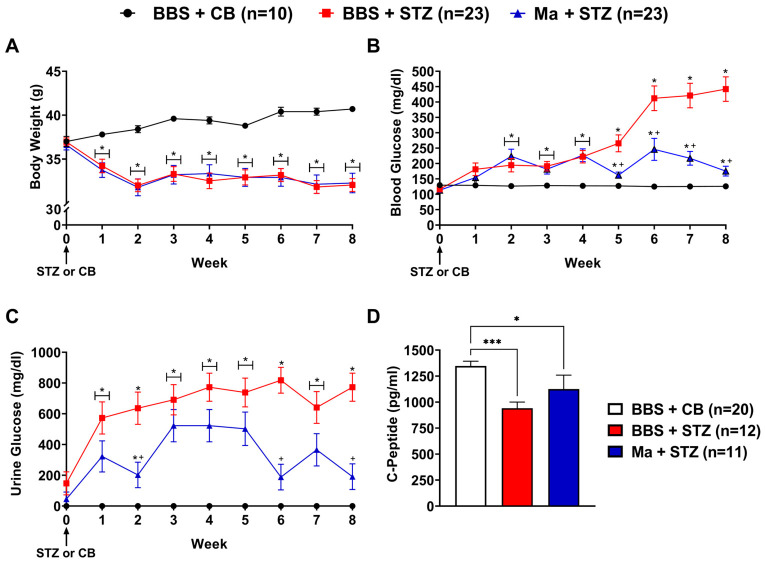
Effect of oral prophylactic administration of HK *M. aurum* on body weight, *C*-peptide, blood and urine glucose levels in STZ-induced diabetic mice. Three different groups of BALB/c mice were orally administered with 3 doses of borate-buffered saline (BBS) or HK *M. aurum* (Ma; 1 mg per injection) given 2 weeks apart. After 6 weeks (at week 0), diabetes was induced in 2 groups of mice [(BBS + STZ) and (Ma + STZ) groups] through injecting them with a single dose of STZ (150 mg/kg). The third group (BBS + CB) received citrate buffer (CB) instead of STZ and served as the vehicle-treated, non-diabetic group. Mice (**A**) body weights, (**B**) fasting blood glucose levels, and (**C**) urine glucose levels were analyzed on a weekly basis as of week 0 up to week 8 post-CB/STZ injection, whereas (**D**) serum *C*-peptide levels were only recorded at week 8 post-CB/STZ injection. (**A**–**C**) Each symbol represents the mean value ± SEM of body weight, blood or urine glucose level for each mouse group. (**D**) Column bars represent the mean value ± SEM of serum *C*-peptide level for each mouse group. Statistically significant differences between the mean body weights, blood and urine glucose levels of different mouse groups were determined by two-way ANOVA followed by Tukey post hoc test. Statistically significant differences between the mean serum *C*-peptide levels of different mouse groups were determined by one-way ANOVA followed by Fisher LSD post hoc test. * *p* < 0.05 and *** *p* < 0.001 versus BBS + CB group; ^+^ *p* < 0.05 versus BBS + STZ group; ├┤: includes both BBS + STZ and Ma + STZ groups.

We next examined whether prophylactic oral administration of HK *M. aurum* can prevent the weight loss normally noted among STZ-induced diabetic mice. Both untreated (BBS + STZ) and *M. aurum*-treated (Ma + STZ) diabetic mouse groups exhibited a similar pattern of body weights over the 8-week period post-diabetes induction by STZ injection (Figure 1A). However, the average body weights of the aforementioned groups were significantly (*p* < 0.05) lower (<35 g) than that of the normal non-diabetic group during the study period, whereby mice in the normal group displayed an increase in their body weights (Figure 1A). According to Figure 1B, non-diabetic mice (BBS + CB) displayed normal blood glucose levels that ranged from 125 to 130 mg/dL throughout the 8-week period post-CB injection. On the other hand, STZ injection significantly (*p* < 0.05) elevated blood glucose levels in untreated (BBS + STZ) and *M. aurum*-treated groups (Ma + STZ) as of week 2 post-STZ injection and by week 4, both groups became diabetic (blood glucose levels > 200 mg/dL) (Figure 1B). However, diabetic mice pre-treated with 3 oral doses of HK *M. aurum* (Ma + STZ) exhibited blood glucose levels that were significantly (*p* < 0.05) lower by 39%, 40%, 48%, and 60% than those of untreated diabetic mice (BBS + STZ) at weeks 5, 6, 7 and 8 post-STZ injection, respectively (Figure 1B). Despite this substantial reduction, blood glucose levels in the *M. aurum*-treated group remained above 200 mg/dL at weeks 6 and 7 of the study period, indicating persistent hyperglycemia relative to the non-diabetic control group (BBS + CB; <200 mg/dL). We next studied urine glucose levels in different groups of mice. Glucose was undetectable in the urine of non-diabetic control mice (BBS + CB), whereas untreated (BBS + STZ) and *M. aurum*-treated (Ma + STZ) diabetic mice exhibited a rapid increase in urinary glucose concentrations from week 1 through week 8 post-STZ injection (Figure 1C). Mice that received three oral administrations of HK *M. aurum* (Ma + STZ) prior to diabetes induction by STZ exhibited lower urinary glucose levels than those observed in vehicle-treated diabetic mice (BBS + STZ); however, these differences were statistically significant only at weeks 2, 6, and 8 (Figure 1C). To indirectly evaluate the efficiency of pancreatic β-cells to produce insulin, serum *C*-peptide levels were measured across experimental groups. *C*-peptide concentrations were significantly (*p* < 0.001) reduced in the diabetic (BBS + STZ) group (941 ± 59.5 pg/mL) compared to the non-diabetic control (BBS + CB) group (1347 ± 46 pg/mL), thus confirming β-cell dysfunction induced by STZ (Figure 1D). Mice prophylactically treated with *M. aurum* exhibited a trend (*p* > 0.05) toward higher *C*-peptide levels (1126 ± 113 pg/mL) relative to the untreated diabetic (BBS + STZ) group. However, *C*-peptide levels in the *M. aurum*-treated group remained significantly (*p* < 0.05) lower than those of the non-diabetic control group (Figure 1D).

### 3.2. Oral Prophylactic Treatment of Diabetic Mice with HK M. aurum Does Not Avert the Dysregulated Expression of SM and Hepatic Proteins Involved in Glucose Transport, Glycolytic Activity, Mitochondrial Uncoupling, and Antioxidant Defense

**Figure 2:**
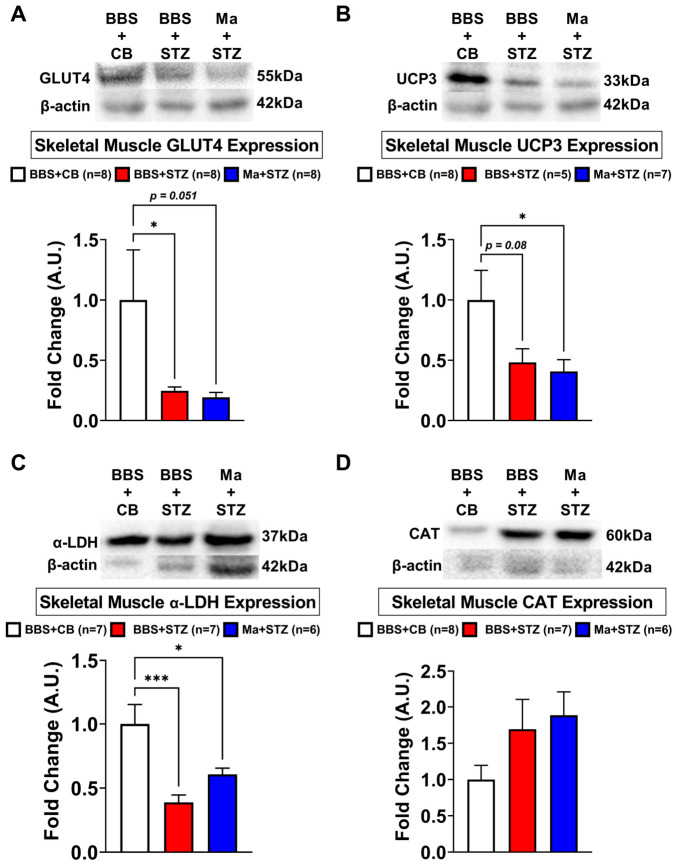
Effect of oral prophylactic administration of HK *M. aurum* on skeletal muscle GLUT4, UCP3, α-LDH, and CAT protein expression in STZ-induced diabetic mice. Three different groups of BALB/c mice were orally administered with 3 doses of borate-buffered saline (BBS) or HK *M. aurum* (Ma; 1 mg per injection) given 2 weeks apart. After 6 weeks (at week 0), diabetes was induced in 2 groups of mice [(BBS + STZ) and (Ma + STZ) groups] through injecting them with a single dose of STZ (150 mg/kg). The third group (BBS + CB) received citrate buffer (CB) instead of STZ and served as the vehicle-treated, non-diabetic group. (**A**) GLUT4, (**B**) UCP3, (**C**) α-LDH, and (**D**) CAT protein expression levels were determined in the skeletal muscles of different mouse groups at week 8 post-CB/-STZ injection by Western blot, as shown in the representative immunoblot (upper panel). Column bar graphs (lower panel) present the results of quantitative densitometric analysis (arbitrary units, A.U.), with protein levels normalized to β-actin and expressed as fold change relative to the non-diabetic control group (BBS + CB). Data are presented as mean ± SEM. Group comparisons were performed using one-way ANOVA followed by Fisher’s LSD post hoc test. Differences in protein expression between groups were considered statistically significant at * *p* < 0.05 and *** *p* < 0.001.

To identify potential molecular correlates of the observed reduction in hyperglycemia following the prophylactic oral administration of HK *M. aurum* to diabetic mice, we examined the expression of proteins involved in glucose transport (GLUT2, GLUT4 and SGLT2), glycolytic activity (α-LDH), mitochondrial uncoupling (UCP2 and UCP3), and antioxidant defense (CAT) in various tissues. GLUT4, the major insulin-sensitive glucose transporter in SM, was significantly (*p* < 0.05) reduced in the SM of the untreated diabetic group compared to non-diabetic controls (Figure 2A). This reduction (<2-fold) was also noted in the *M. aurum*-treated group, whereby it did not reach statistical significance (*p* = 0.051); however, no major difference was observed between BBS + STZ and Ma + STZ groups (Figure 2A). SM GLUT4 expression remained reduced in *M. aurum*-treated diabetic mice to a similar extent as in untreated diabetic mice, with no significant difference between the two diabetic groups. SM UCP3, which is involved in mitochondrial energy regulation, was also downregulated in both untreated and *M. aurum*-treated diabetic mice as compared to non-diabetic mice; however, this downregulation attained statistical significance only in the untreated diabetic mouse group (Figure 2B). In a similar manner, SM α-LDH expression levels were comparable between untreated (BBS + STZ) and *M. aurum*-treated (Ma + STZ) diabetic mice; however, they were both significantly (*p* < 0.05) lower than non-diabetic controls (BBS + CB) (Figure 2C). In contrast, there was a trend towards elevated SM CAT expression in untreated diabetic mice (BBS + STZ) as well as in diabetic mice prophylactically treated with *M. aurum* (Ma + STZ) in comparison to healthy control mice (BBS + CB) (Figure 2D).

**Figure 3:**
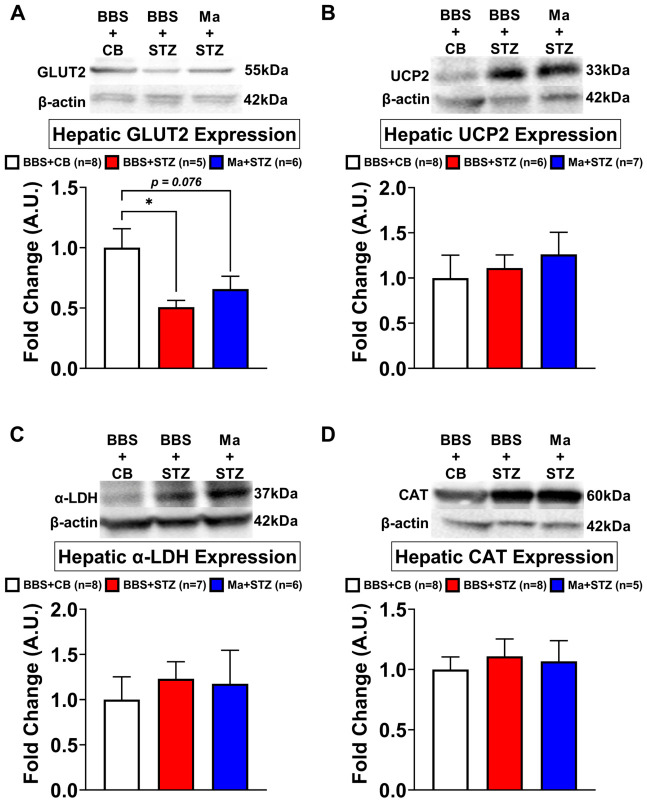
Effect of oral prophylactic administration of HK *M. aurum* on hepatic GLUT2, UCP2, α-LDH, and CAT protein expression in STZ-induced diabetic mice. Three different groups of BALB/c mice were orally administered with 3 doses of borate-buffered saline (BBS) or HK *M. aurum* (Ma; 1 mg per injection) given 2 weeks apart. After 6 weeks (at week 0), diabetes was induced in 2 groups of mice [(BBS + STZ) and (Ma + STZ) groups] through injecting them with a single dose of STZ (150 mg/kg). The third group (BBS + CB) received citrate buffer (CB) instead of STZ and served as the vehicle-treated, non-diabetic group. (**A**) GLUT2, (**B**) UCP2, (**C**) α-LDH, and (**D**) CAT protein expression levels were determined in the livers of different mouse groups at week 8 post-CB/STZ injection by Western blot, as shown in the representative immunoblot (upper panel). Column bar graphs (lower panel) present the results of quantitative densitometric analysis (arbitrary units, A.U.), with protein levels normalized to β-actin and expressed as fold change relative to the non-diabetic control group (BBS + CB). Data are presented as mean ± SEM. Group comparisons were performed using one-way ANOVA followed by Fisher’s LSD post hoc test. Differences in protein expression between groups were considered statistically significant at * *p* < 0.05.

We next assessed the impact of HK *M. aurum* oral prophylactic treatment on GLUT2, UCP2, α-LDH, and CAT protein expression levels in the liver of diabetic mice. Oral prophylactic treatment of mice with HK *M. aurum* (Ma + STZ) did not prevent the STZ-induced reduction in the hepatic expression of the insulin-independent glucose transporter, GLUT2, as its level was not significantly different from that observed in untreated diabetic mice (BBS + STZ). However, hepatic GLUT2 expression level in both groups of mice was lower than that of the non-diabetic control group (BBS + CB) (Figure 3A). Moreover, Western blot analysis revealed a trend towards a modestly enhanced expression of hepatic UCP2 (Figure 3B), α-LDH (Figure 3C), and CAT (Figure 3D) proteins in both untreated (BBS + STZ) and prophylactically *M. aurum*-treated (Ma + STZ) diabetic mice.

**Figure 4:**
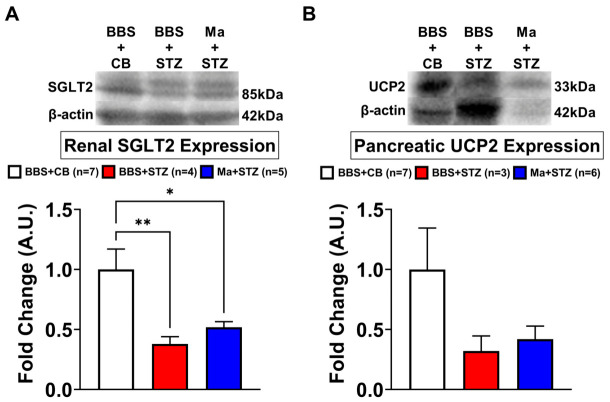
Effect of oral prophylactic administration of HK *M. aurum* on renal SGLT2 and pancreatic UCP2 protein expression in STZ-induced diabetic mice. Three different groups of BALB/c mice were orally administered with 3 doses of borate-buffered saline (BBS) or HK *M. aurum* (Ma; 1 mg per injection) given 2 weeks apart. After 6 weeks (at week 0), diabetes was induced in 2 groups of mice [(BBS + STZ) and (Ma + STZ) groups] through injecting them with a single dose of STZ (150 mg/kg). The third group (BBS + CB) received citrate buffer (CB) instead of STZ and served as the vehicle-treated, non-diabetic group. (**A**) Renal SGLT2 and (**B**) pancreatic UCP2 protein expression levels were determined in different mouse groups at week 8 post-CB/-STZ injection by Western blot, as shown in the representative immunoblot (upper panel). Column bar graphs (lower panel) present the results of quantitative densitometric analysis (arbitrary units, A.U.), with protein levels normalized to β-actin and expressed as fold change relative to the non-diabetic control group (BBS + CB). Data are presented as mean ± SEM. Group comparisons were performed using one-way ANOVA followed by Fisher’s LSD post hoc test. Differences in protein expression between groups were considered statistically significant at * *p* < 0.05 and ** *p* < 0.01.

Given that SGLT2 plays a critical role in renal glucose reabsorption, we examined its expression in the kidneys of different mouse groups. As shown in Figure 4A, there was a significant (*p* < 0.01) decrease (~2.6-fold) in renal SGLT2 protein expression in the diabetic mouse group (BBS + STZ), as compared to the normal mouse group (BBS + CB). Moreover, diabetic mice pretreated orally with HK *M. aurum* (Ma + STZ) exhibited a non-significantly higher level of renal SGLT2 expression than untreated diabetic mice; however, its expression remained significantly lower than that of normal mice (Figure 4A). In pancreatic β-cells, UCP2 modulates mitochondrial oxidative phosphorylation and regulates insulin release [22]. Accordingly, pancreatic UCP2 expression was reduced in diabetic mice compared to non-diabetic controls, but this reduction did not reach statistical significance. Oral pretreatment with *M. aurum* (Ma + STZ) failed to prevent this decline, as pancreatic UCP2 levels were comparably reduced (Figure 4B). These findings are best considered exploratory, and increased biological replication (*n* > 5) specifically in the BBS + STZ group would be required to confidently assess the effects of HK *M. aurum* on renal and pancreatic protein expression.

### 3.3. Therapeutic ID Administration of HK M. aurum Ameliorates Hyperglycemia in STZ-Induced Diabetic Mice

**Figure 5:**
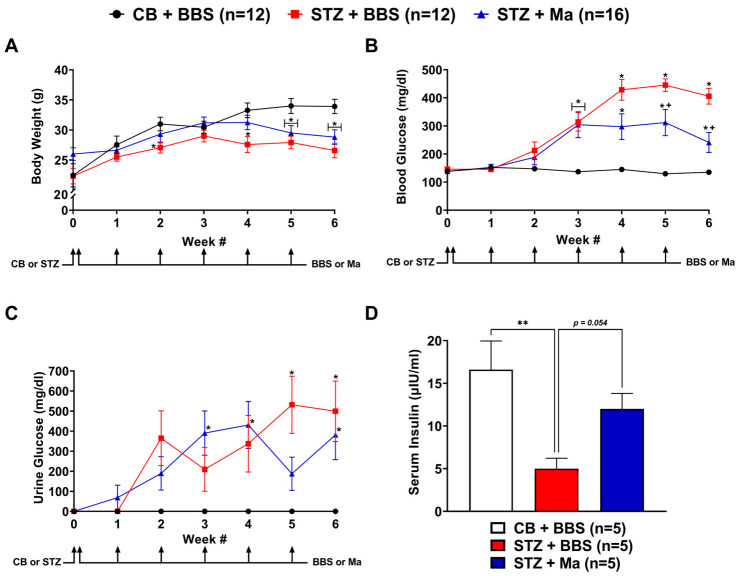
Effect of intradermal therapeutic administration of HK *M. aurum* on body weight, insulin, blood and urine glucose levels in STZ-induced diabetic mice. BALB/c mice were divided into three groups: Group 1 (CB + BBS), which served as the vehicle-treated, non-diabetic group was injected with citrate buffer (CB) and treated with 6 doses of borate buffered saline (BBS); Group 2 (STZ + BBS) was injected with STZ (150 mg/kg) and treated with 6 doses of BBS, Group 3 (STZ + Ma) was injected with STZ (150 mg/kg) and treated with 6 doses of HK *M. aurum* (1 mg/injection). Treatment with BBS or HK *M. aurum* was given on a weekly basis as of day 1 post-CB/-STZ injection and over a period of 5 weeks. Mice (**A**) body weights, (**B**) fasting blood glucose levels, and (**C**) urine glucose levels were analyzed on a weekly basis up to week 6 post-CB/-STZ injection. (**D**) Mice serum insulin levels were only measured at week 6 post-CB/-STZ injection. (**A**–**C**) Each symbol represents the mean value ± SEM of body weight, blood or urine glucose level for each mouse group. (**D**) Column bars represent the mean value ± SEM of serum insulin level for each mouse group. Statistically significant differences between the mean body weights, blood and urine glucose levels of different mouse groups were determined by two-way ANOVA followed by Tukey post hoc test. Statistically significant differences between the mean serum insulin levels of different mouse groups were determined by one-way ANOVA followed by Fisher LSD post hoc test. * *p* < 0.05 and ** *p* < 0.01 versus BBS + CB group; ^+^ *p* < 0.05 versus BBS + STZ group; ├┤: includes both BBS + STZ and Ma + STZ groups. # indicates the week number of the experiment.

As a preliminary safety assessment, we administered six ID doses of HK *M. aurum* to normal non-diabetic mice and evaluated potential adverse effects, focusing on alterations in body weight and fasting blood glucose concentrations. In a previous study by our group, ID prophylactic treatment of non-diabetic mice with 3 doses of HK *M. aurum* (1 mg/injection), given every 2 weeks, did not result in changes in the body weight, blood glucose, and urine glucose levels of mice [13]. In the current study, weekly measurements (over a period of 6 weeks) of body weight and blood glucose levels (Supplementary Figure S4) did not demonstrate significant differences between vehicle-treated (CB + BBS) and HK *M. aurum*-treated (CB + Ma) non-diabetic mice. Both groups of mice displayed a similar trend in terms of weight gain over the 6-week period. Moreover, both groups exhibited comparable normal (<200 mg/dL) blood glucose levels (Supplementary Figure S4). The second arm of our study examined the potential anti-diabetic activity of HK *M. aurum* through restoring normal weight gain and lowering hyperglycemia and glycosuria in STZ-induced diabetic mice. Mice injected with a single high dose of STZ were later treated with 6 doses of HK *M. aurum* given on a weekly basis and starting 24 h post-STZ injection. Normal non-diabetic mouse group (CB + BBS) exhibited a steady increase in its body weight from week 1, and this increase reached ~51% by week 6 (Figure 5A). Conversely, the untreated diabetic mouse group (STZ + BBS) showed a slight increase (~20%) in body weight; however, its body weight at weeks 4–6 was significantly (*p* < 0.05) lower than that of the normal group (Figure 5A). The body weight of the diabetic mouse group treated with HK *M. aurum* (STZ + Ma) was comparable to that of the untreated diabetic group and was significantly lower than that of the non-diabetic group at weeks 5 and 6 (Figure 5A). The fasting blood glucose level of the control non-diabetic group was normal (<200 mg/dL), whereby it ranged between 130 and 160 mg/dL over the 6-week period (Figure 5B). In the untreated STZ-induced diabetic group (STZ + BBS), hyperglycemia was detected as of week 3 post-STZ injection with a significant (*p* < 0.05) increase noted at weeks 4–6, with the mean glucose levels of this group being maintained above 400 mg/dL (Figure 5B). Intriguingly, administration of HK *M. aurum* to STZ-induced diabetic mice (STZ + Ma) resulted in a trend towards attenuated hyperglycemia in this group after the fourth treatment dose (at week 4 post-STZ injection), as compared to untreated STZ-induced diabetic mice (STZ + BBS). However, a statistically significant (*p* < 0.05) 30% and 40% reduction in blood glucose levels of *M. aurum*-treated diabetic group was observed following the fifth (at week 5 post-STZ injection) and sixth (at week 6 post-STZ injection) treatment doses, respectively (Figure 5B). Nevertheless, blood glucose levels in the “STZ + Ma” group remained >200 mg/dL, indicating partial rather than complete glycemic correction. While no trace of glucose was detected in the urine of the normal vehicle group (CB + BBS), significant (*p* < 0.05) detectable glucose levels were noted in the urine of the untreated STZ-induced diabetic group at weeks 3–6 post-STZ injection (Figure 5C). Treatment of the STZ-induced diabetic group with HK *M. aurum* (STZ + Ma) failed to significantly diminish glycosuria in this group, though there was a trend towards lower urine glucose levels at weeks 5 and 6, as compared to the untreated STZ-diabetic group (Figure 5C). As expected, serum insulin level significantly (*p* < 0.05) declined at week 6 by ~3.3-fold in the untreated diabetic group (STZ + BBS) when compared with that of the normal non-diabetic group (CB + BBS) (Figure 5D). However, treatment of diabetic mice with HK *M. aurum* seemed to improve insulin secretion in this group (STZ + Ma), which exhibited a trend towards higher serum insulin level than those of the untreated diabetic group (STZ + BBS) (Figure 5D).

### 3.4. Therapeutic ID Administration of HK M. aurum Restores the Expression of SM UCP3, Hepatic UCP2, and α-LDH in STZ-Induced Diabetic Mice

**Figure 6:**
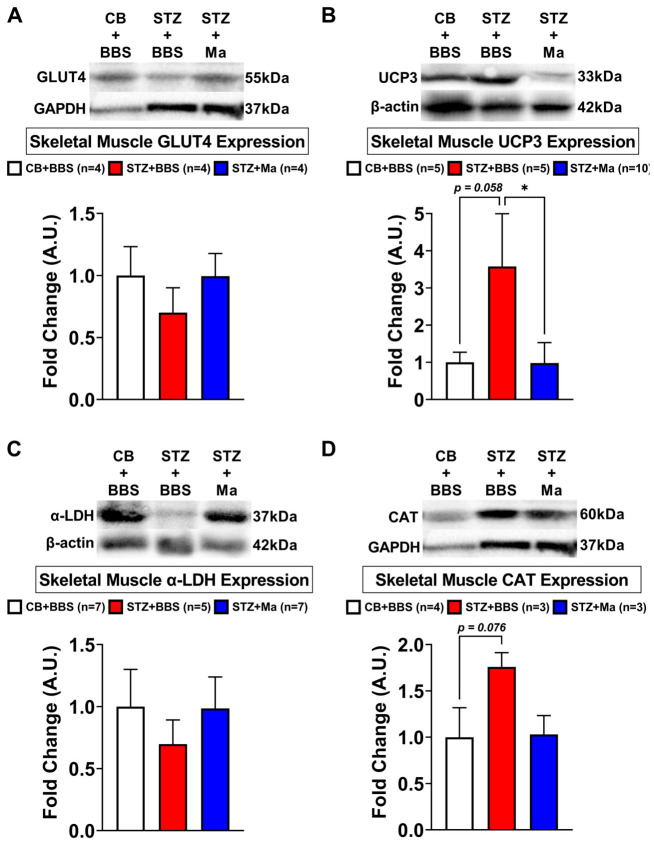
Effect of intradermal therapeutic administration of HK *M. aurum* on skeletal muscle GLUT4, UCP3, α-LDH, and CAT protein expression in STZ-induced diabetic mice. BALB/c mice were divided into three groups: Group 1 (CB + BBS), which served as the vehicle-treated, non-diabetic group was injected with citrate buffer (CB) and treated with 6 doses of borate buffered saline (BBS); Group 2 (STZ + BBS) was injected with STZ (150 mg/kg) and treated with 6 doses of BBS, Group 3 (STZ + Ma) was injected with STZ (150 mg/kg) and treated with 6 doses of HK *M. aurum* (1 mg/injection). Treatment with BBS or HK *M. aurum* was given on a weekly basis as of day 1 post-CB/STZ injection and over a period of 5 weeks. (**A**) GLUT4, (**B**) UCP3, (**C**) α-LDH, and (**D**) CAT protein expression levels were determined in the skeletal muscles of different mouse groups at week 6 post-CB/-STZ injection by Western blot, as shown in the representative immunoblot (upper panel). Column bar graphs (lower panel) present the results of quantitative densitometric analysis (arbitrary units, A.U.), with protein levels normalized to β-actin or GAPDH and expressed as fold change relative to the non-diabetic control group (BBS + CB). Data are presented as mean ± SEM. Group comparisons were performed using one-way ANOVA followed by Fisher’s LSD post hoc test. Differences in protein expression between groups were considered statistically significant at * *p* < 0.05.

Western blotting was employed to determine whether therapeutic administration of HK *M. aurum* modulates proteins central to metabolic regulation, including glucose transporters (GLUT2 and GLUT4), a glycolytic enzyme (α-LDH), mitochondrial uncoupling proteins (UCP2 and UCP3), and the antioxidant enzyme, CAT. We subsequently determined the expression levels of these proteins in SM and liver tissues of the distinct experimental mouse groups. For SM GLUT4, the sample size was limited to four mice per group (Figure 6A). Within this constraint, no statistically significant changes were noted between the vehicle group (CB + BBS) and the untreated diabetic group (STZ + BBS), although the latter showed a trend toward reduced GLUT4 expression. Treatment of diabetic mice with HK *M. aurum* resulted in a trend toward upregulation of GLUT4, but this did not achieve statistical significance. Accordingly, these GLUT4 trends should be interpreted with caution.

Given the vital protective roles of mitochondrial UCPs against mitochondrial dysfunction and oxidative stress, we questioned whether the expression levels of UCP3 were dysregulated in the SM of STZ-induced diabetic mice, and whether HK *M. aurum* can correct for such an abnormal expression if present. STZ-induced diabetic mice showed an almost significant (*p* = 0.058) ~3.5-fold increase in SM UCP3 expression levels compared with those detected in the SM of non-diabetic vehicle-injected mice (Figure 6B). Therapeutic administration of HK *M. aurum* to STZ-induced diabetic mice significantly reduced the STZ-induced increase in SM UCP3 expression, resulting in a ~3.7-fold significant (*p* < 0.05) downregulation in SM UCP3 expression, which was comparable to that of non-diabetic mice (Figure 6B). A non-significant trend toward higher SM CAT expression was observed in untreated diabetic mice (STZ + BBS) compared with non-diabetic controls (CB + BBS) (*p* = 0.076). Therapeutic ID administration of HK *M. aurum* to diabetic mice (STZ + Ma) appeared to normalize SM CAT expression; however, this reversal did not reach statistical significance, possibly due to limited sample size (*n* = 3–4 mice/group) (Figure 6D).

**Figure 7:**
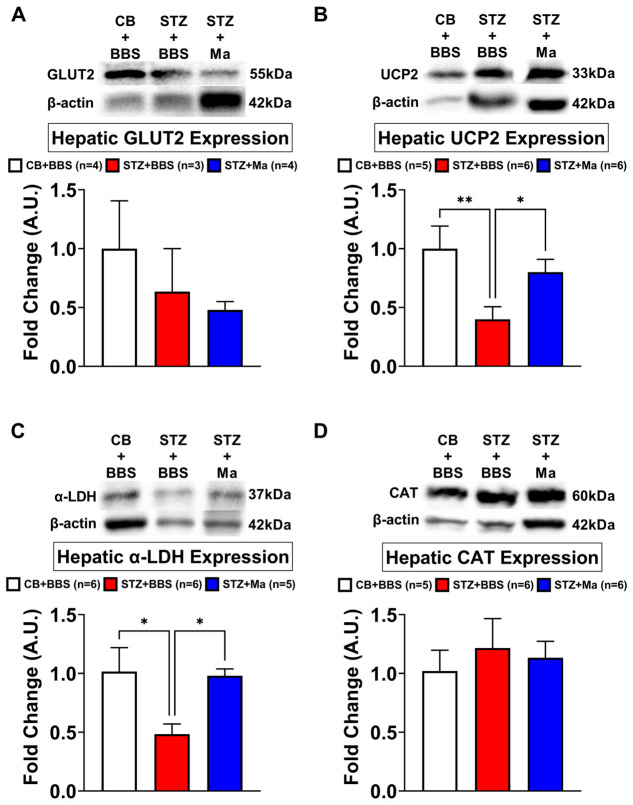
Effect of intradermal therapeutic administration of HK *M. aurum* on hepatic GLUT2, UCP2, α-LDH, and CAT protein expression in STZ-induced diabetic mice. BALB/c mice were divided into three groups: Group 1 (CB + BBS), which served as the vehicle-treated, non-diabetic group was injected with citrate buffer (CB) and treated with 6 doses of borate buffered saline (BBS); Group 2 (STZ + BBS) was injected with STZ (150 mg/kg) and treated with 6 doses of BBS, Group 3 (STZ + Ma) was injected with STZ (150 mg/kg) and treated with 6 doses of HK *M. aurum* (Ma; 1 mg/injection). Treatment with BBS or HK *M. aurum* was given on a weekly basis as of day 1 post-CB/-STZ injection and over a period of 5 weeks. (**A**) GLUT2, (**B**) UCP2, (**C**) α-LDH, and (**D**) CAT protein expression levels were determined in the livers of different mouse groups at week 6 post-CB/-STZ injection by Western blot, as shown in the representative immunoblot (upper panel). Column bar graphs (lower panel) present the results of quantitative densitometric analysis (arbitrary units, A.U.), with protein levels normalized to β-actin or GAPDH and expressed as fold change relative to the non-diabetic control group (BBS + CB). Data are presented as mean ± SEM. Group comparisons were performed using one-way ANOVA followed by Fisher’s LSD post hoc test. Differences in protein expression between groups were considered statistically significant at * *p* < 0.05 and ** *p* < 0.01.

Hepatic GLUT2 expression showed no statistically significant differences between any experimental groups (Figure 7A). However, the sample size for this analysis was limited (*n* = 3–4 per group), which reduces the ability to detect modest differences. Therefore, these data do not rule out biologically relevant effects of HK *M. aurum* on hepatic GLUT2. Untreated diabetic mice exhibited a pronounced and significant (*p* < 0.001) suppression in liver UCP2 protein expression, showing a ~2.5-fold decrease relative to non-diabetic controls. Following ID administration of six doses of HK *M. aurum*, diabetic mice demonstrated a significant (*p* < 0.005) 2-fold elevation in liver UCP2 expression when compared with their untreated counterparts, effectively normalizing UCP2 levels to those seen in non-diabetic mice (Figure 7B). As illustrated in Figure 7C, untreated diabetic mice exhibited a pronounced (*p* < 0.05) decline in their liver α-LDH expression levels, falling to nearly half of those measured in the healthy control mice (Figure 7C). Notably, treatment of diabetic mice with HK *M. aurum* for six weekly doses counteracted this reduction, normalizing α-LDH expression and yielding a ~50% significant (*p* < 0.05) increase compared with the untreated diabetic group (Figure 7C). Finally, comparison of experimental groups showed that liver CAT expression remained essentially unchanged between normal mice (CB + BBS) and untreated diabetic mice (STZ + BBS), despite a small trend toward higher levels in the latter. Moreover, giving diabetic mice six doses of HK *M. aurum* (STZ + Ma) did not lead to detectable alterations in liver CAT expression (Figure 7D).

## 4. Discussion

Our previous work has demonstrated that ID prophylactic administration of HK *M. aurum* to mice alleviates hyperglycemia following a single injection of a high STZ dose (150 mg/kg), suggesting a protective effect on glucose regulation [13]. Building on this finding, the current work examined whether HK *M. aurum* can similarly reduce hyperglycemia via two alternative, but complementary strategies, prophylactic oral administration and therapeutic intradermal delivery, while also assessing effects on glucose handling across key metabolic tissues in STZ-induced diabetic BALB/c mice.

The current findings strongly support the classification of HK *M. aurum* as a potential postbiotic, a preparation of an inanimate microorganism that possesses a health benefit on the host, according to the International Scientific Association of Probiotics and Prebiotics (ISAPP) consensus definition [23]. Unlike viable probiotics, postbiotics exhibit better safety, shelf stability, and ease of incorporation into functional food matrices. Given that HK *M. aurum* is already commercially available as an oral dietary supplement, its potential as a functional food ingredient for glycemic management is potentially translatable, but only after confirmation in more clinically relevant animal models and human trials. Recent studies have demonstrated that other inanimate microorganisms, such as pasteurized *A. muciniphila* and HK *Lacticaseibacillus rhamnosus*, improve insulin sensitivity and lower blood glucose in both animal models and human trials [11,24,25]. Our findings add to this literature, but remain correlative, not mechanistic.

Data from the present study demonstrated that oral prophylactic and intradermal therapeutic administration of HK *M. aurum* markedly alleviated hyperglycemia in STZ-induced diabetic mice, although *M. aurum*-treated diabetic mice remained hyperglycemic. Reductions in blood glucose reached ~39% and ~60% at weeks 5 and 8 post-STZ injection, respectively. This mycobacteria-mediated anti-hyperglycemic effect indicates improved systemic glucose regulation, highlighting the robust biological activity of HK *M. aurum*. *C*-peptide reflects endogenous insulin production, and maintenance of residual β-cell reserve has been linked to improved long-term outcomes in T1D and T2D [26,27]. The noted trend towards improved serum *C*-peptide secretion in mice prophylactically treated with *M. aurum* raises the hypothesis, but does not prove, that HK *M. aurum* may preserve residual β-cell function. Moreover, early ID therapeutic administration of HK *M. aurum* after STZ-induced diabetes resulted in a moderate, yet significant, reduction in hyperglycemia (~30% at week 5; ~40% at week 6) and was associated with a trend toward enhanced insulin secretion (~58% increase), yet blood glucose levels remained above normal. The observed differences in glycemic outcomes between the prophylactic and therapeutic arms should be interpreted with caution, as the two arms also differed in route of administration, dosing frequency, and endpoint timing. While timing relative to diabetes onset is one contributing factor, the study design does not allow us to isolate its specific contribution. Therefore, comparisons between arms are primarily hypothesis-generating. The prophylactic oral arm showed numerically greater glycemic reduction than the therapeutic intradermal arm; however, because multiple variables differed between arms (route, frequency, and endpoint), we cannot conclude that prophylactic administration is inherently more effective. One possible explanation, which requires confirmation in a controlled comparative study, is that earlier intervention before established oxidative and inflammatory injury confers greater benefit. This aligns with prior evidence showing that pretreatment with Bacillus Calmette-Guérin (BCG), a live attenuated vaccine derived from *Mycobacterium bovis*, reduces hyperglycemia in STZ-induced diabetic mice [28,29]. It is speculated that once STZ-mediated β-cell destruction, mitochondrial dysfunction, NF-κB activation, and reactive oxygen species (ROS)-driven damage are established, reversal is challenging, which likely explains the attenuated, yet still significant, anti-hyperglycemic effects observed following the therapeutic administration of *M. aurum* to STZ-induced diabetic mice. Equally important, the prophylactic and therapeutic anti-hyperglycemic efficacy of HK *M. aurum* was not accompanied by off-target metabolic effects such as induction of hypoglycemia or alteration in body weight in healthy non-diabetic mice, indicating metabolic specificity. Our findings further corroborate the previously reported safety profile of HK *M. aurum* in animals [15]. While our findings demonstrate partial anti-hyperglycemic effects in a β-cell dysfunction state (single-injection, high-dose STZ model), extrapolation to other forms of diabetes, particularly T2D, should be approached cautiously and would require direct validation in appropriate models such as high-fat-diet-fed low-dose STZ-injected mice, *db*/*db*, and *ob*/*ob* mice.

To elucidate the mechanisms underlying HK *M. aurum*-mediated glycemic control in STZ-induced diabetic mice, we investigated protein expression levels of glucose transporters (GLUT2, GLUT4, and SGLT2), metabolic enzymes (α-LDH), ROS modulators (UCP2 and UCP3), and antioxidant defenses (CAT) across mouse tissues in both arms of the study. Across metabolically active tissues, HK *M. aurum* elicited distinct yet coordinated effects on mitochondrial regulation, underscoring the tissue-specific nature of metabolic adaptation in the STZ model. In the therapeutic arm, untreated STZ-injected mice exhibited significant downregulation of hepatic UCP2 and α-LDH. Moreover, HK *M. aurum* treatment of STZ-injected mice normalized the expression of α-LDH and UCP2 to near-normal levels. This finding is consistent with, but does not prove, enhanced mitochondrial function. Therefore, functional studies such as the direct measurement of mitochondrial oxygen consumption rates, ATP synthesis, or ROS production are required. In contrast, in the prophylactic arm, hepatic α-LDH and UCP2 levels remained comparable to non-diabetic controls following STZ exposure, indicating no measurable mitochondrial suppression under this setting. Accordingly, HK *M. aurum* preventive treatment did not further alter their expression. This highlights the context-dependent nature of STZ, whose biological effects are influenced by factors such as dose variability, age, body weight, inflammatory status, and timing of tissue sampling, all of which modulate hepatic responses to treatment [30].

In our prophylactic model, untreated diabetic mice exhibited a marked downregulation of pancreatic UCP2. Reduced UCP2 expression has been linked to impaired mitochondrial redox balance and increased β-cell oxidative injury [31], while others have described such changes in the context of inflammatory stress [32,33]. Therefore, lower pancreatic UCP2 expression in STZ-diabetic mice could reflect altered mitochondrial adaptation, but whether this translates to reduced β-cell resilience or increased vulnerability remains to be established. SM exhibited context-dependent mitochondrial responses to STZ, reflected by differential regulation of UCP3 across experimental cohorts. In the therapeutic ID model, STZ-induced hyperglycemia was accompanied by upregulation of UCP3. Previous studies have linked increased UCP3 expression to compensatory mitochondrial uncoupling under oxidative stress [34,35]; however, direct functional measurements are vital to determine whether such a mechanism occurs in our model. ID HK *M. aurum* administration normalized UCP3 expression, suggesting attenuation of excessive uncoupling. In contrast, STZ exposure in the prophylactic model resulted in downregulation of SM UCP3. This divergence indicates that factors beyond exposure time, such as route of administration, systemic distribution, and baseline metabolic state, critically shape SM mitochondrial responses. Given UCP3’s sensitivity to fatty acid flux and oxidative stress [35], variability in STZ composition or metabolic status can shift mitochondrial signaling and lead to opposing regulation patterns [36]. Oral prophylactic administration of HK *M. aurum* did not prevent reduced SM UCP3 expression, despite significantly lowering blood glucose. These findings illustrate the diabetogenic nature of STZ, which can drive either induction or suppression of UCP3 depending on metabolic context, and highlight that HK *M. aurum*’s effects on UCP3 expression are tissue- and context-dependent.

Hepatic CAT expression did not exhibit significant variations between groups, providing no evidence for altered antioxidant enzyme expression in the liver. In SM, CAT expression showed non-significant trends whereby a tendency toward higher levels in untreated diabetic mice (prophylactic arm) and a trend toward normalization in *M. aurum*-treated diabetic mice (therapeutic arm) were noted. However, without direct measurements of ROS or oxidative damage, these CAT expression trends cannot be interpreted as evidence for changes in mitochondrial stress, ROS generation, or redox balance. Thus, observed patterns paralleled UCP3 expression changes in some cohorts, but this correlation does not establish a causal or functional link between mitochondrial uncoupling and antioxidant responses. Accordingly, direct oxidative stress assays are required to determine whether HK *M. aurum* affects redox status. Building on tissue-specific protein expression patterns related to mitochondrial and redox pathways, our glucose transporter analyses provide preliminary, correlative evidence that *M. aurum*’s metabolic benefits may operate independently of insulin-dependent glucose transport. Hepatic GLUT2 expression remained suppressed in treated diabetic mice, consistent with impaired hepatic glucose sensing [37], while SM GLUT4 was reduced in the oral prophylactic cohort and unchanged in the ID therapeutic cohort. Similarly, renal SGLT2 expression was decreased in untreated diabetic mice and unaltered by prophylactic treatment.

We acknowledge that protein expression analyses, mainly in the therapeutic arm, for SM GLUT4, SM CAT, and hepatic GLUT2 were derived from very small sample sizes (*n* = 3–4 mice per group). These specific results are preliminary and inconclusive, and they should not be interpreted as supporting any mechanistic conclusion unless replicated in larger cohorts. Other analyses, such as SM UCP3 and hepatic UCP2/α-LDH, had slightly larger sample sizes (*n* = 5–10 mice per group), lending more confidence to those correlative findings. Nevertheless, all protein expression data are correlative by nature, whereby they demonstrate associations, but do not establish causation. Therefore, the observed *M. aurum*-mediated partial anti-hyperglycemic effects cannot be attributed directly to alterations in these proteins without functional validation. Given these limitations, the data are best viewed as hypothesis-generating. They are consistent with the hypothesis that HK *M. aurum* might influence systemic metabolism through pathways that may involve mitochondrial or redox-related proteins. However, direct measurements, such as mitochondrial respiration, ROS production, insulin sensitivity, or β-cell function, are required to evaluate such hypotheses.

Postbiotic functional foods are gaining attention, with supplementation shown to reduce insulin, triglycerides, and inflammatory markers [38]. From a functional food perspective, the oral prophylactic data are most relevant, demonstrating partial prevention of severe hyperglycemia in an STZ-induced model. It remains to be determined whether similar effects occur in more translationally relevant models and if the underlying mechanisms align with those proposed for BCG vaccination in T1D [29,39]. Additional studies using HK *M. aurum*-treated STZ-induced diabetic mice are needed to define the immune pathways linking microbial sensing to glycemic control, including cytokine networks and PRR signaling. Parallel investigations should assess epigenetic modifications associated with trained immunity. Additionally, examining mitochondrial dynamics and biogenesis will be particularly important to determine whether the observed changes in UCP2/UCP3 translate into altered mitochondrial function. At the pancreatic level, histological analyses will be essential to assess whether any β-cell preservation reflects reduced apoptosis or enhanced proliferation. Finally, gut microbiome profiling in the oral administration model may clarify microbiota–host contributions to *M. aurum*’s metabolic effects.

## 5. Conclusions

Collectively, this work supports the concept that inactivated mycobacteria can influence glucose regulation in a high-dose STZ-induced diabetes mouse model and identifies HK *M. aurum* as a candidate worthy of further investigation for its potential to achieve partial glycemic improvement in insulin-deficient states. Direct comparison of prophylactic versus therapeutic efficacy of HK *M. aurum* treatment is precluded by differences in route, dosing frequency, and experimental timeline. Our findings provide a correlative basis for further investigation. Therefore, functional studies are currently needed to determine whether HK *M. aurum* directly modulates mitochondrial uncoupling, oxidative stress, or insulin-independent glucose disposal pathways.

## Data Availability

The original contributions presented in this study are included in the article/Supplementary Material. Further inquiries can be directed to the corresponding author.

## Supplementary Materials

The following supporting information can be downloaded at: https://www.mdpi.com/article/10.3390/nu18111652/s1, Figure S1. Experimental design for evaluating the prophylactic anti-diabetic potential of heat-killed *Mycobacterium aurum* oral administration in streptozotocin-induced diabetic BALB/c mice. Figure S2. Experimental design for evaluating the therapeutic anti-diabetic potential of heat-killed *Mycobacterium aurum* intradermal administration in streptozotocin-induced diabetic BALB/c mice. Figure S3. Effect of oral prophylactic administration of HK *M. aurum* on body weight and blood glucose levels of non-diabetic mice. Figure S4. Effect of intradermal therapeutic administration of HK *M. aurum* on body weight and blood glucose levels of non-diabetic mice.
