# Supplementary material for: Prophylactic and Therapeutic Anti-Hyperglycemic Effects of Heat-Killed Mycobacterium aurum in STZ-Induced Diabetic Mice
Source: Nutrients. 2026 May 22;18(11):1652. doi: 10.3390/nu18111652 (PMC13258161; doi:10.3390/nu18111652)
Supplement: Supplementary file 1 [file nutrients-18-01652-s001.zip › nutrients-4283841-supplementary.pdf]

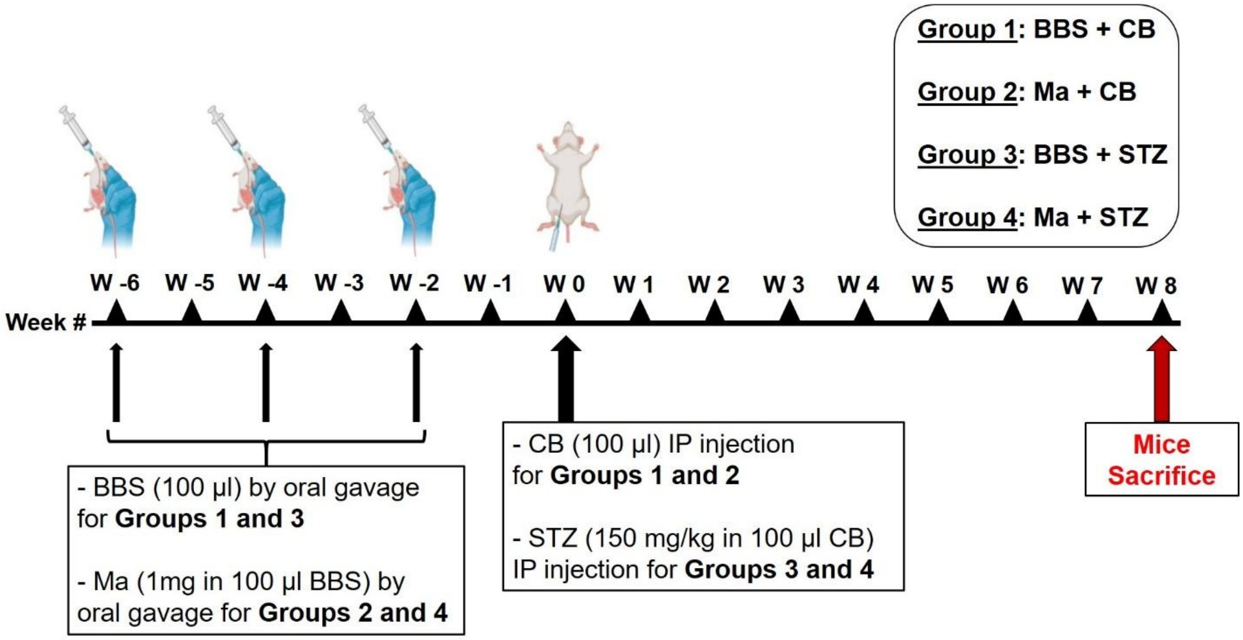

Supplementary Figure S1 Experimental design for evaluating the prophylactic anti-diabetic potential of heat-killed *Mycobacterium aurum* oral administration in streptozotocin-induced diabetic BALB/c mice. borate buffered saline (BBS); citrate buffer (CB); intraperitoneal (IP); *Mycobacterium aurum* (Ma); streptozotocin (STZ).

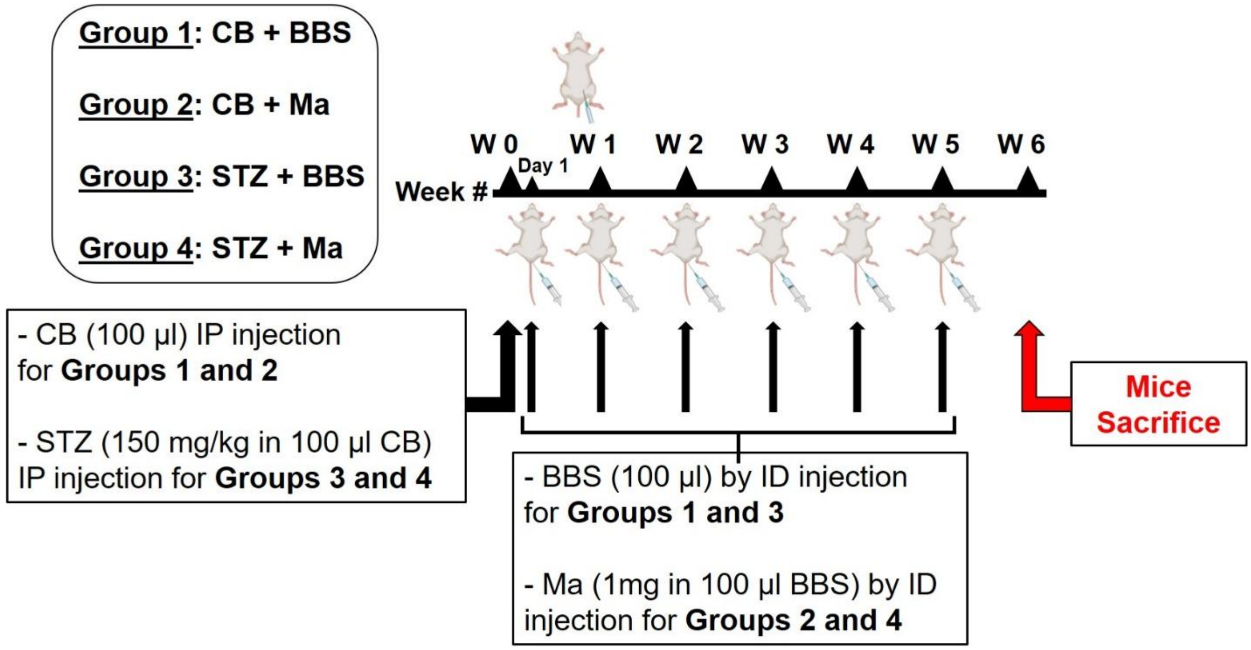

Supplementary Figure S2. Experimental design for evaluating the therapeutic anti-diabetic potential of heat-killed *Mycobacterium aurum* intradermal administration in streptozotocin-induced diabetic BALB/c mice. borate buffered saline (BBS); citrate buffer (CB); intradermal (ID); intraperitoneal (IP); *Mycobacterium aurum* (Ma); streptozotocin (STZ).

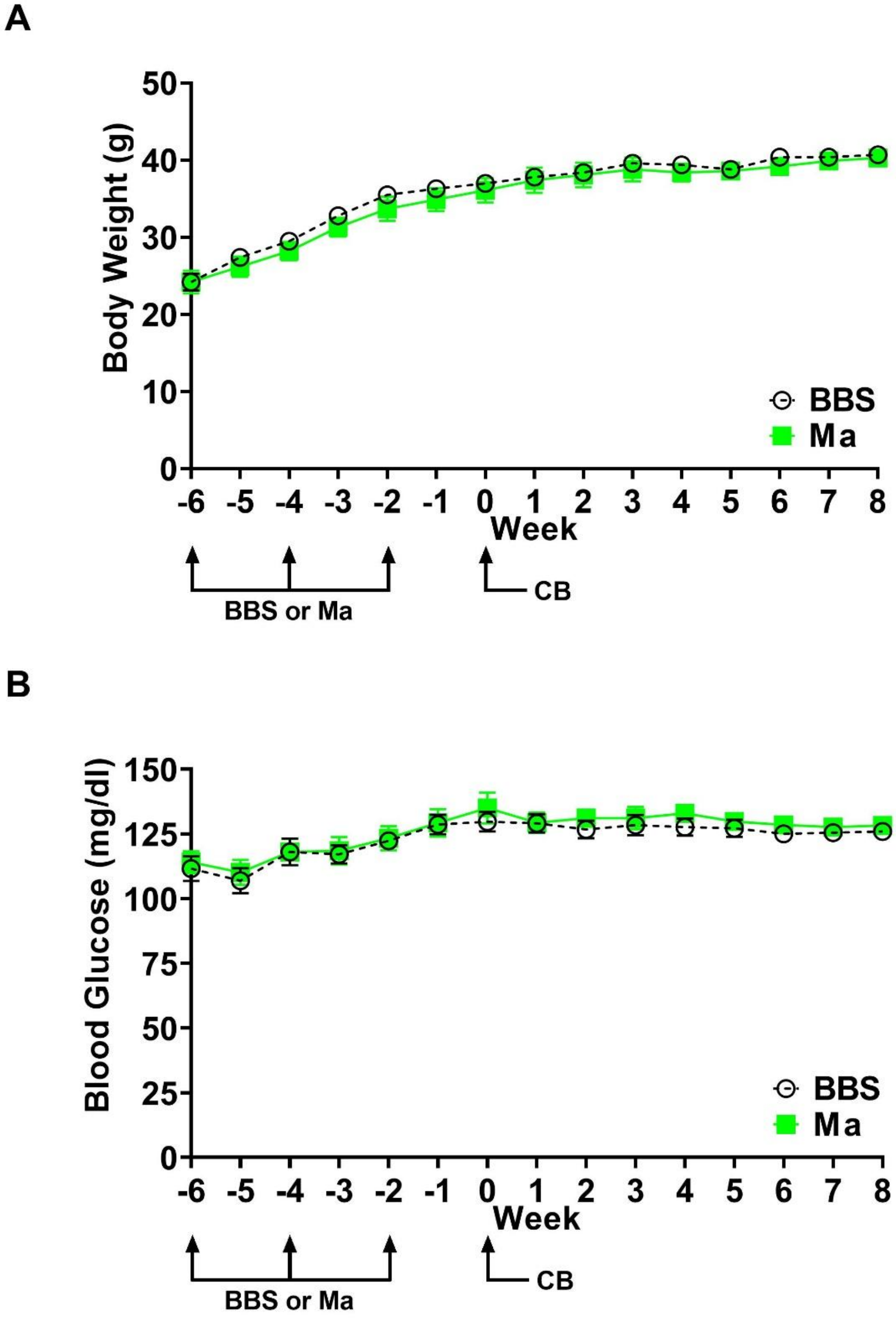

Supplementary Figure S3. Effect of oral prophylactic administration of HK *M. aurum* on body weight and blood glucose levels of non-diabetic mice. Two different groups of BALB/c mice were orally administered with 3 doses of borate-buffered saline (BBS) or HK *M. aurum* (Ma; 1 mg per injection) given 2 weeks apart. After 6 weeks (at week 0), mice received citrate buffer (CB). Mice (A) body weights, and (B) fasting blood glucose levels were analyzed on a weekly basis from week -6 to week 8 post-CB injection. (A–B) Each symbol represents the mean value ± SEM of body weight and blood glucose level for each mouse group (n = 10 mice/group).

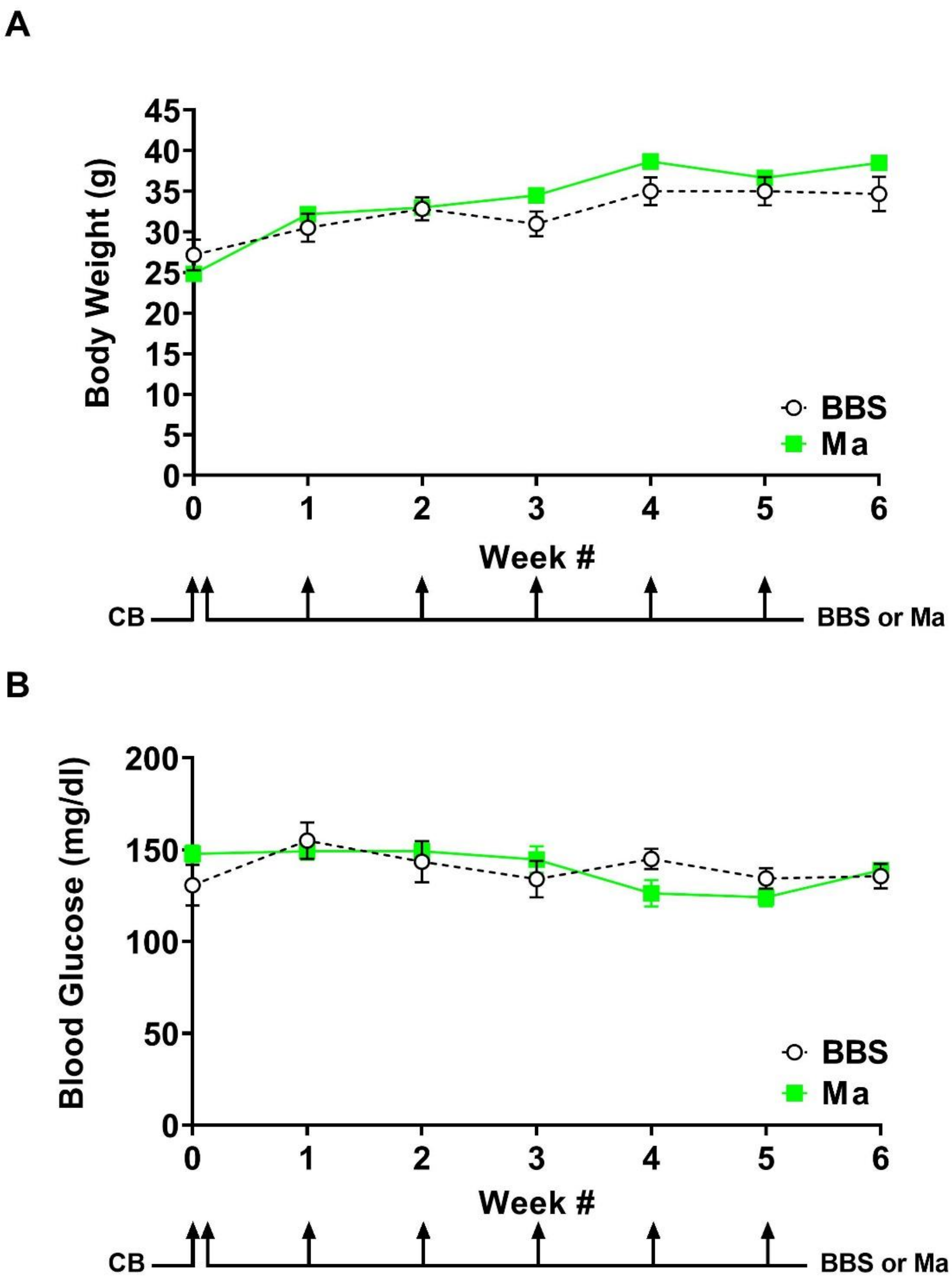

Supplementary Figure S4. Effect of intradermal therapeutic administration of HK *M. aurum* on body weight and blood glucose levels of non-diabetic mice. BALB/c mice were divided into two groups: Group 1 (CB+BBS), which served as the vehicle-treated non-diabetic group, was injected with citrate buffer (CB) and treated with 6 doses of borate buffered saline (BBS); Group 2 (CB+Ma) was injected with CB and treated with 6 doses of HK *M. aurum* (Ma; 1 mg/injection). Treatment with BBS or Ma was given on weekly basis as of day 1 post-CB injection and over a period of 5 weeks. Mice (A) body weights and (B) fasting blood glucose levels were analyzed on weekly basis up to week 6 post-CB injection. (A–B) Each symbol represents the mean value ± SEM of body weight or blood glucose level for each mouse group (n = 6 mice/group).
